# Exercise intensity during exergaming in wheelchair-dependent persons with SCI

**DOI:** 10.1038/s41393-023-00893-3

**Published:** 2023-04-03

**Authors:** Matthijs Ferdinand Wouda, Jon-Arve Gaupseth, Espen Ingvald. Bengtson, Truls Johansen, Espen Andreas Brembo, Eivind Lundgaard

**Affiliations:** 1 Sunnaas Rehabilitation Hospital, Nesoddtangen, Norway; 2 Centre for Health and Technology, University of South-Eastern Norway (USN), Drammen, Norway

**Keywords:** Spinal cord diseases, Metabolism

## Abstract

**Study design:**

Cross-sectional study.

**Objectives:**

It is challenging for persons with SCI, especially those who are wheelchair dependent, to find suitable and motivating aerobic exercise modes. Exergaming might be a good option, since it is relatively cheap and can be played at home, alone or with others. However, it is unknown if exergaming is performed at a sufficient exercise intensity.

**Setting:**

Sunnaas Rehabilitation Hospital, Norway.

**Methods:**

Twenty-two men and two women (*n* = 24) with chronic SCI (AIS A-C), all wheelchair users, were included during inpatient rehabilitation. All participant performed a maximal graded arm-crank test (pretest), while measuring peak oxygen uptake (VO_2peak_) and peak heart rate (HR_peak_). The day after they had a practice playing session with three different exergames (X-box Kinect, Fruit Ninja; Nintendo Wii, Wii Sports Boxing; VR Oculus Rift, boxing). The following day, participants played each exergame for 15 min. During these 45 min of exergaming, exercise intensity, based on VO_2peak_ and HR_peak_ from the pretest, was monitored.

**Results:**

Approximately 30 of the 45 min of exergaming was performed at moderate or high intensity. Participants exercised on average 24.5 min (95%CI 18.7–30.5) at moderate intensity (>50–80% VO_2peak_) and 6.6 min (95%CI 2.2–10.8) at high intensity (>80% VO_2peak_).

**Conclusions:**

The participants were able to exercise at moderate or high intensity during exergaming in a considerable amount of time. Exergaming seems to be suitable for aerobic exercise at an intensity that can provide health benefits in wheelchair-dependent persons with SCI.

## Introduction

Persons with spinal cord injury (SCI) constitutes a high-risk group for developing an inactive and sedentary lifestyle with an accelerated trajectory of secondary adverse health conditions similar to those found in the general population but with even more devastating outcomes [1]. A person’s physical activity level after a SCI depends on the severity of the injury, i.e., injury level [2, 3]. Compared to healthy persons, daily energy expenditure is significantly lower in people with SCI, due to a lack of motor function, and fewer opportunities to engage in physical activity [4]. In general, the wheelchair depended part of the SCI population is most inactive and show the lowest levels of cardiorespiratory fitness (CRF) [5]. CRF is the capacity of the human circulatory and respiratory systems to supply oxygen to skeletal muscles for energy production needed during physical activity [6]. The plurality of wheelchair-dependent people with SCI is in need for strategies to increase physical activity levels, with the intention to increase CRF and reduce the risk of cardiovascular disease [4, 7, 8].

To counter these negative trends, exercise guidelines for persons with SCI have been developed [9]. These guidelines recommend at least 20 min with aerobic exercise at moderate or high intensity, twice a week, to achieve health benefits. However, there are many barriers to everyday physical activity for persons with SCI, such as accessibility of stores and buildings and physical constrains [10]. Many of the available wheelchair-specific exercise modes are experienced as little motivating, especially when performed over time [11]. Therefore, it might be challenging for persons with SCI, especially those who are wheelchair dependent, to find suitable and motivating exercise modes.

There is no universal definition of exergaming. Gao et al. describe exergames as digital games that require bodily movements to play, stimulating an active gaming experience to function as a form of physical activity [12]. Exergames require digital devices, such as computers or game consoles and their accessories, such as virtual reality (VR) goggles. Exergaming might be a good alternative exercise mode for wheelchair users, since it is relatively cheap and can be played at home, alone or with others. Exergaming is often experienced as more motivating compared to other, more traditional exercise modes [13].

To promote exergaming as an exercise mode to achieve the recommendations for physical activity for wheelchair users [9], it is necessary to determine the exercise intensity during exergaming. Several studies have described oxygen uptake (VO_2_) or metabolic equivalent of task (i.e., absolute exercise intensity) during exergaming in wheelchair dependent persons with SCI [11, 14]. These studies showed low to moderate exercise intensity during exergaming. However, absolute exercise intensities are less applicable in individuals with physical disabilities, as their physical constraints might limit them to reach a high absolute exercise intensity. In these cases, the relative exercise intensity, i.e., exercise intensity described in percentage of a persons’ peak oxygen consumption (%VO_2peak_) or peak heart rate (%HR_peak_) is a better alternative. Based on heart rate (HR) or VO_2_ levels, exercise intensity can be divided into *low* (<70 %HR_peak_ or <50% VO_2peak_), *moderate* (between 70 and 85 %HR_peak_ or between 50 and 80 % VO_2peak_) and *high* intensity (>85 %HR_peak_ or >80% VO_2peak_) [15].

To the best of our knowledge, only two other studies, both with rather few participants, have described exercise intensity during exergaming in percentage of peak VO_2_ or peak HR [16, 17] in persons with neurological disabilities. Most of the participants in these studies achieved moderate exercise intensity (>50% VO_2peak_). However, it is challenging to interpret these findings without taking into account how other factors, such as injury-related characteristics, participants’ physical fitness level and type of game, influence exercise intensity during exergaming in this SCI subpopulation.

Thus, in the present study, we aim to explore the relative exercise intensity (% VO_2peak_ and % HR_peak_) during exergaming in wheelchair dependent persons with SCI, subsequent to a maximal graded test. Secondary aims are to evaluate if type of game, CRF and the level of SCI influence the ability of the participants to execute exergaming at moderate and high intensity.

## Methods

### Design

This cross-sectional study has included wheelchair-dependent persons with SCI, from the Department of Spinal Cord Injury at Sunnaas Rehabilitation Hospital in Norway.

#### Participants

**Fig. 1:**
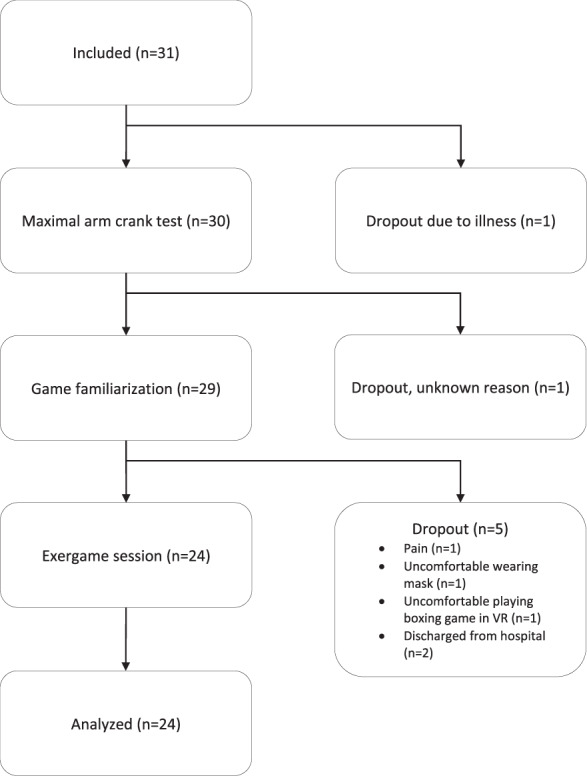
Flowchart of the study, including dropouts.

All of the 31 patients that were asked to participate in this study were recruited over a 2-year period (2018–2020). Due to a dropout of seven patients, a total of 24 participants (22 men and 2 women) were included for data analysis (see Fig. 1). Inclusion criteria were SCI injury level C5 or lower (AIS A-C), ≥1 year post-injury, age ≥18 years and being dependent of a wheelchair. For the purpose of this study, ‘wheelchair dependent’ was defined as not being able to actively stand in an upright position without external support.

Participants were excluded if they had concurrent medical conditions that might limit their CRF (e.g., psychiatric conditions, orthopedic diseases, or uncontrolled cardiopulmonary disease).

#### Procedures

The study was approved by the Norwegian Centre for Research Data (Ref.: 962676). All participants gave written informed consent before inclusion. After medical approval for inclusion, the participants performed a maximal exercise test on a stationary arm cycle at the Clinical Physiological Laboratory. The following day, the participants came back to the laboratory where they became familiar with the games used in this study. This supervised practice training session lasted for ~45 min. 2–3 days after, the participants performed the exergame session. All participants played three different games for 15 min each, with a 5 min rest in between sessions. The three games were played at different game consoles: Fruit Ninja (Xbox Kinect (Microsoft Xbox™, Microsoft, Redmond, WA, USA)), Boxing (Nintendo Wii (Nintendo™, Kyoto, Japan)) and VR boxing (Oculus Rift (Oculus™, Meta, Menlo Park, CA, USA)). Fruit Ninja (Xbox Kinect) is controlled by body motions, being picked up and analyzed by a camera. The two other games, Wii Sports Boxing (Nintendo Wii) and Thrill of the Fight (Oculus Rift), both boxing games, are played by imitating boxing movements while holding two movement sensitive game controllers.

The order of the games was randomized through block randomization by drawing numbered lots. All subjects were asked to refrain from alcohol, exercise or strenuous physical activity 12 h prior to the exercise test and the exergame session.

#### Maximal graded test

The participants were instructed to perform a five-minute warm-up on an electrically braked arm cycle ergometer (LODE, Groningen, The Netherlands) at 20 watts with a crank rate of 60 revolutions per minute (RPM), while sitting in their own wheelchair. During the maximal exercise test, the initially workload was set to 20 watt for 3 min and was thereafter individually adjusted with 10–40 watt each third minute, until volitional exhaustion [18]. The hands of participants with poor hand function were attached to the handlebars using specially designed gloves. Oxygen uptake and heart rate were measured throughout the test. The highest measured VO_2_ (average over 30 s) was considered as VO_2peak_. The highest measured HR during this test was considered HR_peak_. Two minutes after termination of the test, a blood sample was taken from the fingertip to measure blood lactate.

#### Exergame session

The exergame session started with collecting 5 min of resting data, in which the participants were asked to sit as quietly as possible in their wheelchair. Thereafter, the participants played a series of three different games for 15 min per game with a 5 min rest in between. For those who had experienced reduced sitting balance during the familiarization session, a Velcro band was attached around their chest and the back of the wheelchair. When necessary, the gaming controller was taped to the person’s hands. The wheelchair was placed on and attached to a low platform with straps. Prior to the session, the participants were instructed to envision exergaming as an exercise mode. They received no motivational feedback during exergaming. VO_2_ and HR were measured throughout the session.

#### Outcome measures

VO_2_ was measured by using a computerized standard open-circuit technique breath-by-breath spirometer (Vmax 220, Sensormedics Corporation, Yorba Linda, CA, USA) during both the maximal graded exercise test and the exergame session. In addition, respiratory exchange ratio (RER), HR (Polar M400), and blood lactate [La-] (BIOSEN C-line, Sport, EFK diagnostics, Barleben, Germany) were measured to evaluate whether criteria for maximal exercise testing were achieved. Achievement of VO_2peak_ was verified by fulfilling three (or four) the following criteria: (1) A plateau in VO_2_ (<2 ml/kg/min increase despite increased workload), (2) Blood lactate concentration ≥8 mmol [La^−1^], (3) Respiratory exchange ratio ≥1.15, (4) Ninety % of age-predicted maximal HR [19]. We used the formula 211-0.64*age for age-predicted maximal HR for exercise testing on a treadmill as proposed by Nes et al [20]. However, the expected maximal heart rate during arm cycling is 90% of the HR achieved during treadmill running [15]. Therefore we used the following adjusted formula to calculate each participant’s age-predicted maximal heart rate during arm cranking: (211-0.64*age)*0.9.

#### Data analysis

Tables and figures have been created in Microsoft Excel (Microsoft Corperation, 2016). Exercise intensity, expressed by % VO_2peak_ and % HR_peak_ were calculated individually, based on the VO_2_ and HR responses at maximal effort, during the maximal graded test. Comparisons of achieved exercise intensity during the different exergames and the influence of CRF and injury level was based on VO_2_ measurements, not HR. The rationale is that % VO_2_ peak is suggested as a more valid method to estimate exercise intensity than HR [18].

Statistical analyses were performed with SPSS (IBM Corp. Released 2021. IBM SPSS Statistics for Windows, Version 28.0. Armonk, NY: IBM Corp). Data are reported as mean and standard deviation (SD) unless otherwise stated. For all tests, statistical significance was set at an alpha level of 0.05. To compare exercise intensities during the three different exergames, repeated measures ANOVA was calculated followed by a post-hoc Fisher’s least significant difference (LSD) test to determine which groups were different from each other. Linear regression analysis was used, with VO_2peak_ as independent factor, to identify possible differences in exercise intensity based on the participants’ CRF level. Finally, Independent-samples Mann–Whitney U test was used to analyze the between-group difference of participants with a cervical and thoracic/lumbar SCI.

## Results

### Participants

Table 1 describes participants’ demographics, spinal cord injury-specific characteristics and their cardiovascular responses achieved at maximal effort during the maximal graded test, both as individual and mean values, including SD. Blood lactate levels for seven participants are however missing due to technical failure.

**Table 1:** Patients’ demographics, injury-specific characteristics and cardiorespiratory responses at maximal effort during the arm-cranking test.

	Demographics			Injury			Peak response to arm-cranking test							
	Age (year)	Sex	BMI (kg/m^2^)	Level of Injury	AIS	Duration (year)	VO_2 peak_ (ml kg^−1^ min^−1^)	VO_2 peak_ (liter min^−1^)	CRF^a^ classification	PO _peak_ (W)	Watt/kg	HR peak	RER	[LA^−1^] (mmol/liter)
1	46–60	Female	14.2	C5	C	45–50	18.3	0.64	Excellent	33	0.94	143	1.10	6.49
2	31–45	Male	21.9	T2	A	16–20	22.5	1.69	Excellent	103	1.37	188	1.44	*Missing*
3	46–60	Male	16.4	T9	A	26–30	23.6	1.32	Excellent	98	1.75	189	1.43	*Missing*
4	16–30	Male	20.7	T3	A	11–15	19.3	1.29	Good	65	0.97	191	1.34	*Missing*
5	16–30	Female	25.5	T11	B	21–25	18.4	1.23	Good	76	1.13	201	1.49	*Missing*
6	46–60	Male	24.7	T3	A	11–15	17.5	1.40	Average	80	1.00	151	1.14	*Missing*
7	31–45	Male	21.1	T8	A	21–25	24.2	1.98	Excellent	120	1.46	172	1.50	13.47
8	46–60	Male	23.8	T4	A	21–25	19.6	1.67	Good	87	1.02	167	1.46	8.66
9	16–30	Male	17.5	C7	A	11–15	27.3	1.72	Excellent	85	1.35	147	1.25	8.89
10	16–30	Male	22.3	C7	A	6–10	16.3	1.12	Excellent	67	0.97	126	1.29	10.54
11	16–30	Male	30.2	T10	A	6–10	24.3	2.62	Excellent	130	1.20	186	1.39	12.54
12	46–60	Male	23.2	T7	A	6–10	35.9	2.55	Excellent	120	1.69	170	1.64	13.99
13	16–30	Male	21.6	C5	A	11–15	14.1	1.04	Good	35	0.47	107	1.20	*Missing*
14	46–60	Male	24.8	T3	A	35–39	17.3	1.31	Average	65	0.86	170	1.25	*Missing*
15	46–60	Male	40.9	T10	A	21–25	15.7	2.15	Average	105	0.77	156	1.42	9.78
16	31–45	Male	31.4	T3	A	11–15	18.4	1.90	Good	100	0.97	177	1.27	9.09
17	61–75	Male	28.7	T12	A	26–30	15.5	1.40	Average	70	0.78	150	1.34	8.75
18	16–30	Male	22.4	C6	A	1–5	14.4	1.08	Good	53	0.71	155	1.31	10.46
19	46–60	Male	31.0	T3	B	31–35	15.5	1.47	Average	70	0.74	170	1.37	8.52
20	31–45	Male	23.6	T4	A	11–15	25.2	2.02	Excellent	108	1.35	182	1.34	9.91
21	61–75	Male	29.9	T1	A	16–20	22.8	2.21	Excellent	125	1.29	160	1.27	11.19
22	61–75	Male	23.5	T2	A	1–5	17.1	1.23	Average	60	0.83	123	1.38	8.96
23	16–30	Male	19.6	C6	C	11–15	21.5	1.51	Excellent	70	1.00	110	1.12	8.11
24	31–45	Male	20.9	T9	B	11–15	18.3	1.28	Good	72	1.03	175	1.78	8.63
**Mean (SD)**	**42 (±13)**		**24.2 (±5.7)**			**18.4 (± 10.9)**	**20.1 (±5.0)**	**1.58 (±0.49)**		**83 (±27)**	**1.07 (±0.31)**	**161 (±25)**	**1.36 (±0.16)**	**9.33 (±1.98)**

Both individual results and mean (±SD) are displayed. Mean averages are displayed in **bold**, in the bottom row.

*SD* standard deviation, *SCI* Spinal Cord Injury, *AIS* American spinal cord association Injury Scale, *VO*_*2*_ oxygen uptake, *BMI* body mass index, *kg* kilogram, *m* meter, *min* minute, *ml* milliliter, *CRF* cardiorespiratory fitness, *PO* power output, *W* Watt, *HR* heart rate, *RER* Respiratory Exchange Ratio, [LA^−^] blood lactate.

^a^Participants VO_2peak_ in ml/kg/minute compared to reference values [18].

The participants were aged between 24 and 71 years. Further, the participants’ injury level ranged from; C5–C8 (*n* = 6), Th1–Th6 (*n* = 10) and Th7–12 (*n* = 8). Nineteen of the 24 participants had a complete injury (AIS A), while five participants had an incomplete injury; AIS B (*n* = 3) or AIS C (*n* = 2). Twenty-one out of 24 participants achieved three or four of the criteria for maximal exercise testing, indicating that most participants reached maximal effort during the maximal graded test. Those three subjects reaching one or two criteria had injury level C5 and C6. Eleven participants were classified as having ‘excellent’ cardiorespiratory fitness (CRF), while seven participants had ‘good’ and six ‘average’ CRF levels.

### Exercise intensity during exergaming

Twenty-three out of 24 participants completed the 45 min exergaming session. Most participants showed signs of exhaustion i.e., heavy breathing, sweating, etc. One participant experienced shoulder pain and stopped after 40 min exergaming. No other adverse advents were detected.

**Fig. 2:**
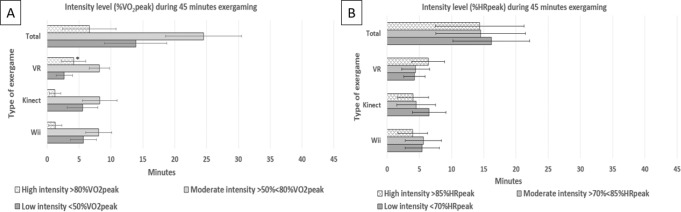
Time spent at low, moderate and high exercise intensity during the 45 min exergaming session. Time spent at low, moderate and high exercise intensity during the 45 min exergaming session, presented as % VO2peak (**A**) and as % HRpeak (**B**), including 95%CI error bars. *HR heart rate (beats per minute), VO*_*2*_ oxygen uptake (milliliter/kilogram/minute). *significant difference *(p* *<* *0.05)*.

Figure 2 shows the mean time participants spent at high, moderate or low intensity, for each of the exergames Fruit Ninja (Microsoft Xbox Kinect), Boxing (Nintendo Wii), and VR boxing (VR Oculus Rift). The achieved exercise intensity during the exergame session is described as % VO_2peak_ (A) and % HR_peak_ (B).

Figure 2A shows that during the 45 min exergame session, participants exercised on average 6.6 min (95%CI 2.2–10.8) at high intensity, 24.5 min (95%CI 18.7–30.5) at moderate intensity and 13.9 min (95%CI 9.0–18.79) at low intensity. Thus, 31 of 45 min with exergaming was performed at moderate or high intensity (>50% VO_2peak_). The duration of exercise spent at moderate intensity did not differ significantly between the different exergames. Post-hoc analysis showed that participants exercised 2.7 min more at high intensity during VR boxing (VR Oculus Rift) compared to Fruit Ninja (Microsoft Xbox Kinect) (*p* = 0.03, 95%CI 0.2–5.2). Otherwise, no significant differences in time spent at high intensity were found between the exergames.

Figure 2B, illustrating exercise intensity in HR peak, shows that participants exercised 14 min (95%CI 7.4–21.2) at high intensity, 14 min (95%CI 7.55–21.5) at moderate intensity and 17 (95%CI 10.3–22.2) min at low intensity. Moreover, 28 of 45 min with exergaming was performed at moderate or high intensity (>70% HR_peak_).

### CRF level

**Fig. 3:**
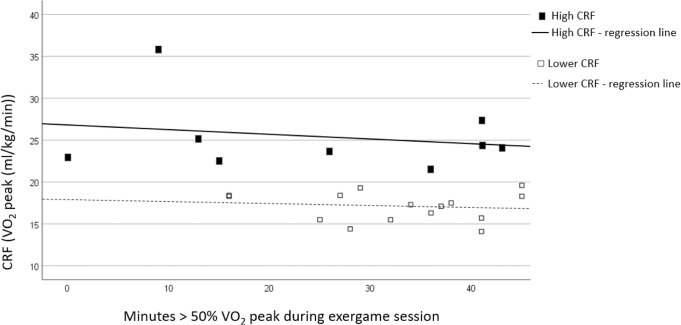
Scatterplot showing the correlation between the participants’ CRF and the time (minutes) spent >50% VO_2peak_. *Participants are divided in two subgroups,* i.e.*, ‘high CRF’ (VO*_*2peak*_ > *20* *ml/kg/min) and ‘lower CRF’ (VO*_*2peak*_ < *20* *ml/kg/min).* *CRF Cardiorespiratory Fitness, VO*_*2peak*_ *peak oxygen uptake*.

Figure 3 shows to which extent the participants’ CRF level affects their ability to exercise at moderate intensity (or more) during the 45 min exergame session. Participants (*n* = 24) are divided in two subgroups, i.e., ‘high CRF’ and ‘lower CRF’, with a cutoff at 20 ml/kg/min, based on the study of Simmons et al. [21].

Eighteen of the 24 participants exercised more than 20 min at an exercise intensity that was >50% VO_2peak_. Nine participants had a VO_2peak_ ≥ 20 ml/kg/min (‘high’ CRF), while the remaining 15 participants had a ‘lower’ CRF level. Participants with lower CRF level exercised on average 32.6 (±9.2) over 50% VO_2peak_, against 25.9 (±16.1) min in those participants with high CRF level. Linear regression analysis showed no significant difference between the high CRF and lower CRF group in the duration that participants exercised >50% of their VO_2peak_ (*p* = 0.09), nor in the duration they exercised >80% of their VO_2peak_ (*p* = 0.82).

### Injury level

**Fig. 4:**
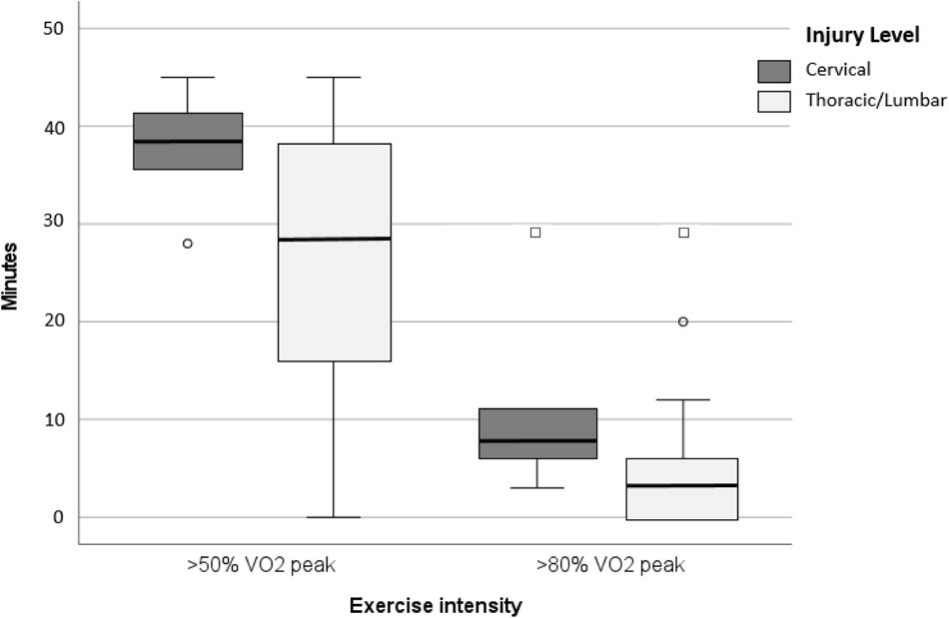
Differences in the time spent at certain exercise intensities (>50% VO_2peak_ and >80% VO_2peak_), between participants with cervical and thoracic/lumbar SCI. □ extreme outlier (Q1–3 * IQR or Q3 + 3 * IQR) [20]. ^*0*^ mild outlier (Q1–1.5 * IQR or Q3 + 1.5 * IQR) [20].

Another secondary aim was to evaluate if the participants’ level of SCI influenced the participants ability to execute exergaming at moderate and high intensity. Figure 4 shows differences in the time spent (minutes) >50% VO_2peak_ and >80% VO_2peak_), between participants with cervical and thoracic/lumbar SCI. Due to skewness of the data, a boxplot (with median and interquartile range) visualizes the between-group differences.

During the 45 min exergame session, participants with a cervical SCI exercised on average 37.8 min (95%CI 31.6–44.0) over 50% VO_2peak_, while participants with lower injury levels exercised 27 min (95%CI 20.5–33.5) 50% VO_2peak_. Participants with a cervical SCI exercised 10.6 min (95%CI 0.9–20.5) over 80% VO_2peak_, while participants with lower injury levels exercised 5.6 min (95% CI 1.7–9.5) 80% VO_2peak_. No significant between-group differences were found in time spent at >50% VO_2peak_ (*p* = 0.08) nor at 80% VO_2peak_ (*p* = 0.07).

## Discussion

The aim of this study was to explore the relative intensity (% VO_2peak_) during exergaming in wheelchair dependent persons with spinal cord injury. Most of the participants were able to exercise a considerable amount of time at moderate or high exercise intensity (i.e., >50% VO_2peak_) during exergaming. The participants’ ability to attain moderate exercise intensities during exergaming seems not to be dependent of their lesion level and CRF level, nor the type of exergame or game console they played.

Participants exercised, on average, 31 min of the 45 min exergaming session with an exercise intensity >50% VO_2peak_. These findings suggest that wheelchair-dependent persons with SCI can meet the SCI recommendations for physical activity of 30 min with aerobic exercise at moderate or high intensity, through 45 min exergaming, twice a week. Thus, exergaming seems to be suitable for aerobic exercise at an intensity that can provide health benefits in wheelchair dependent persons with SCI.

Only a few other studies have performed a VO_2_ peak pre-test to estimate the relative exercise intensity during exergaming (% VO_2peak_) in persons with SCI [16, 17]. In accordance with our results, Widman et al. showed that six out of eight participants with SCI were able to achieve an exercise intensity >50%VO_2peak_ during GameCycle. In the study of Burns et al., all wheelchair dependent participants with SCI (*n* = 9) achieved moderate exercise intensity (>50% VO_2_ reserve) when playing GameCycle Exergaming. However, only 3 out of 9 participants achieved moderate intensity (>50% VO_2_ reserve) when playing XaviX Tennis System exergaming. The type of exergame might therefore play an important role in a person’s ability to achieve moderate or high exercise intensity.

In our study, the achieved exercise time at a moderate intensity was not significantly different when comparing the three exergames/games consoles being used. Nevertheless, an interesting finding was that participants exercised significantly more at high intensity during VR compared to Kinect, but it can be discussed whether a mean difference of 3 min is clinically relevant. The games in the present study were chosen because they were expected to be energy-demanding physical activities. Given the large number of commercial game consoles and games available, it is very well possible that larger differences in exercise intensity between games had been found, if we had selected other games and game consoles.

In the SCI population, the CRF level is dependent on the injury level [21], since VO_2_ peak depends on the amount of intact muscle groups in oxygen need. In our study, neither CRF level nor injury level seem to influence achieved exercise intensity. However, Burns et al. claimed that only persons with a low CRF level would be able to achieve moderate exercise intensity while playing XaviX Tennis System exergaming [16]. Widman et al. states that GameCycle might not be suitable for those with well-developed upper bodies [17]. Therefore, when a certain exergame is played with the purpose of achieving health benefits, it might be advisable to evaluate the exercise intensity, for example with an HR monitor.

Interestingly, there was a large variation in time spent at moderate or high intensity among the participants in our study. One participant did not exceed 50% VO_2peak_, while several others exercised over 40 min >50% VO_2peak_. This indicates that person-dependent factors, such as participants’ interest, motivation and experience with console gaming, may have influenced their exercise intensity during exergaming. Thus, not having assessed these factors is a weakness in the present study and should be included as potentially important variables in future studies.

Although the present study was executed at a hospital, the exergame session was conducted as if it was in a home situation. The staff did not provide verbal encouragements or other motivational expressions to the participants during the exergame session, since this might affect their performance [22, 23]. However, for most participants, the exergames’ in-built visual and audio feedback features were sufficient to attain moderate intensities over a considerable amount of time. Therefore, it is plausible that similar exercise intensities could be achieved in a home setting.

The type of exergames that are commercially available varies widely and can thus meet individual preferences. When exergaming is performed by the user alone in a home setting, surroundings should be ensured, especially when using VR, to avoid injuries. Furthermore, the use of a HR monitor can be considered to evaluate if exergaming is performed at a sufficient exercise intensity level. However, the present study has shown that using a HR monitor might overestimate the time spent at high intensity when compared to exercise intensity expressed as % of VO_2peak_.

One participant experienced shoulder pain throughout the 45 min exergame session. Therefore, untrained wheelchair-dependent persons with SCI should possibly start with shorter exercise sessions, to avoid overuse injuries. Some may therefore need guidance of a local physiotherapist to set up an exercise program, regarding the duration, frequency and intensity of the planned exergame sessions.

In conclusion, exergaming seems to be a promising aerobic exercise mode for wheelchair-dependent persons with SCI. Participants in the present study were able to exercise at a moderate or high exercise intensity level in a considerable amount of time. Based on the findings of this study, we propose that exergaming has potential as an easily accessible and inexpensive exercise mode that can be performed alone or with others at home by persons with SCI. However, future studies with longitudinal design are needed to explore the feasibility and efficiency of a home-based exergame intervention and to determine exercise effects on metabolic health.

## Supplementary information

AJ_checklist

## Data Availability

Additional data are available from the corresponding author on reasonable request.
